# Oxidative stress as a biomarker for monitoring treated celiac disease

**DOI:** 10.1038/s41424-018-0031-6

**Published:** 2018-06-08

**Authors:** Sarah Moretti, Simona Mrakic-Sposta, Leda Roncoroni, Alessandra Vezzoli, Cinzia Dellanoce, Erika Monguzzi, Federica Branchi, Francesca Ferretti, Vincenza Lombardo, Luisa Doneda, Alice Scricciolo, Luca Elli

**Affiliations:** 1 Institute of Bioimaging and Molecular Physiology, National Research Council (CNR), Via Fratelli Cervi 93, 20090 Segrate, Italy; 2 Center for Prevention and Diagnosis of Celiac Disease- Div. of Gastroenterology and Endoscopy, Fondazione IRCCS Ca’Granda Ospedale Maggiore Policlinico, Via F. Sforza 35, 20122 Milano, Italy; 3 Department of Biomedical, Surgical and Dental Sciences, Università degli Studi di Milano, Via Festa del Perdono, 20122 Milano, Italy; 4 Department of Pathophysiology and Transplantation, Università degli Studi di Milano, Via Festa del Perdono, 20122 Milano, Italy; 5 Institute of Clinical Physiology, National Research Council (CNR), Niguarda Ca’ Granda Hospital, Via G. Moruzzi 1, 56124 Pisa, Italy

## Abstract

**Introduction:**

High levels of reactive oxygen species (ROS) and impaired antioxidant defense systems lead to oxidative stress (OxS) and tissue injury in different intestinal and extra intestinal conditions, including celiac disease (CD). The aim of the present study was to investigate the role and potential use of ROS and other biomarkers of OxS in the clinical management of CD.

**Methods:**

We collected duodenal specimens and blood samples from naïve patients (N-CD), patients on a gluten free diet (GFD) including responders (CD-GFD) and non-responders (NRCD).

We measured plasmatic ROS production (electron paramagnetic resonance, EPR), lipid peroxidation (thiobarbituric acid-reactive substances, TBARS), protein oxidation (protein carbonyl, PC), total antioxidant capacity (TAC), nitric oxides and glutathione (GSH) in erythrocytes.

**Results:**

Fifty-four patients affected by CD were enrolled (17 N-CD, 18 CD-GFD and 19 NRCD; 44 F; age 44 ± 13 years). A significant increase of plasmatic OxS biomarkers (ROS, peroxidated lipids, oxidized proteins, and nitrate concentrations) and decrease of antioxidant species (TAC and GSH levels) were found in NRCD and N-CD compared to CD-GFD.

Comparably, a significant direct relationship between the severity of duodenal atrophy, ROS production rates and TBARS was found; conversely, TAC and GSH presented an inverse correlation.

**Discussion:**

OxS is involved in CD tissue damage and correlates with the degree of duodenal atrophy. These findings suggest the possible role of OxS biomarkers as indicators of CD activity during the clinical follow-up.

## Introduction

High levels of reactive oxygen species (ROS) and/or impaired antioxidant defense systems lead to oxidative stress (OxS)^1^ and tissue injury^2^. ROS are produced in cells during the metabolic pathways and they are potentially very dangerous because of their high reactivity. In physiological conditions the deleterious effects of ROS are counteracted by the antioxidant defense systems, such as non-enzymatic antioxidants (glutathione and vitamins) and antioxidant enzymes (i.e., superoxide dismutase, glutathione peroxidase/reductase). If the production of ROS overwhelms the cellular antioxidant capacity, a condition known as OxS occurs^3^.

OxS is implicated in the damage of cellular lipids, proteins, and DNA, increased cellular swelling and decreased cell membrane fluidity. OxS plays an important role in the pathogenesis of many human diseases^4^ including several gastrointestinal disorders^5,6^. In celiac disease (CD), a chronic autoimmune enteropathy triggered by gluten ingestion in genetically predisposed subjects^7^, gluten promotes a Th1-driven autoimmune process that leads to a duodenal mucosal atrophy^8,9^. Currently the only effective treatment normalizing symptoms, autoantibodies (anti-transglutaminase type 2 IgA) and the small bowel mucosa is a strict and chronic gluten-free diet (GFD)^10^.

In most CD patients a clinical response is observed after only a few weeks complying with a GFD treatment^11^. Unfortunately, a complete clinical response and mucosal recovery do not occur in all patients^12^. Indeed, a subgroup of CD patients may have persistent or recurrent symptoms (e.g., diarrhea and abdominal pain), inflammation of the intestine and villous atrophy in spite of their GFD compliance^13^. Non-responsive CD (NRCD) may be defined as the persistence of symptoms, signs, or laboratory abnormalities typical of CD in spite of a 6–12 months long dietary gluten avoidance. NRCD is common, affecting 7–30% of all patients on GFD for CD^14^. There are many distinct etiologies, including unintentional gluten ingestion, other food intolerances (i.e., lactose and fructose), small-intestinal bacterial overgrowth, microscopic colitis, pancreatic insufficiency, irritable bowel syndrome, and refractory CD. While the CD diagnostic criteria are well known and well established, it remains difficult to define a correct use of available biomarkers during follow-up.

The molecular mechanisms underlying CD are still unclear, but a recent in vitro study has shown that OxS is implicated in the pathophysiology of the disease^15–17^. Indeed, several investigations have shown that gluten exposure can induce an intracellular oxidative imbalance in CD patients, characterized by increased levels of lipid peroxidation products and oxidized/reduced glutathione ratio and decreased protein-bound sulfhydryl groups^18^. Moreover, celiac patients have been found to express significantly inducible nitric oxide synthase in the intestinal wall, which results in significantly increased levels of nitric oxide (NO)^19,20^. High contents of NO metabolites were also found in the plasma and serum of untreated CD patient^21,22^.

The broad spectrum of clinical manifestations of CD makes difficult to assess the disease activity in patients on a correct GFD by means of single measurements, while a multidisciplinary approach would possibly generate more meaningful outcome information.

In this regard, the first aim of the study was to investigate the effects of OxS in CD, evaluating the levels of: (i) ROS by using electron paramagnetic resonance (EPR) technique, able to provide the direct detection of the ‘instantaneous’ presence of free radical species in the sample;^23,24^ (ii) oxidative damage biomarkers detected by enzymatic methods in the plasma of celiac patients.

Our secondary aim was to look for new plasma biomarkers corresponding to morphological/functional alterations assessed by histology in duodenal biopsies.

Furthermore, possible correlations between ROS production, other biomarkers of OxS and hematological parameters were also investigated.

## Methods

### Subjects

Duodenal endoscopic biopsies and peripheral blood samples of celiac patients were analyzed. The diagnosis of CD was made at the Center for Prevention and Diagnosis of Celiac Disease of the Fondazione IRCCS Ca’ Granda Ospedale Maggiore Policlinico in Milan (Italy) according to the current international guidelines^6^. Oslo criteria for CD nomenclature has been followed^14^. Fifty-four CD patients was divided into three groups: Naïve celiac patients (N-CD, *n* = 17), GFD-responsive patients (CD-GFD, *n* = 18) and NRCD (*n* = 19). Furthermore, a control group (CTR) was added in the evaluation of ROS production rates, according to the value of ROS production rate assessed in one hundred healthy middle-aged sedentary subjects previously published^24^.

The investigation was approved by the local Ethics Committee (Fondazione IRCCS Ca’ Granda Ospedale Maggiore Policlinico n. 424bis 09/07/2014) and all the patients involved in the study gave their written informed consent.

### Sample preparation

From each patient, at least 5 proximal small-intestinal biopsy specimens, as well as peripheral blood samples, were obtained. Endoscopic duodenal samples were routinely prepared for histopathological analysis as previously described^25^. Venous blood samples were collected, approximately 10 mL in heparinised, and 5 mL in EDTA vacutainer tubes, for the assessment of ROS, OxS biomarkers, hematological parameters and NO metabolites. Blood samples were taken in the morning, after at least 8-hour fasting, by a cubital vein puncture. Hematological values were determined using an automated hematology analyzer (Automatic Beckman Coulter LH-750), according to the standard laboratory procedures.

Parameters, such as hemoglobin (Hgb), hematocrit (Hct), mean corpuscular volume (MCV), mean corpuscular hemoglobin (MCH), mean corpuscular hemoglobin concentration (MCHC), red cell distribution width (RDW), platelets and absolute leukocyte count were recorded.

For the quantification of ROS production and OxS biomarkers, the heparinized blood samples, and for NO metabolites assessment, the EDTA blood samples, were centrifuged at 1000x*g* for 10 min at 4 °C. The samples of plasma and erythrocytes obtained were immediately stored in multiple aliquots at −80 °C until assayed. The samples were thawed only once before analysis, which was carried out within 2 weeks from collection.

### Electron paramagnetic resonance for reactive oxygen species detection

All measurements were carried out by means of a X-band EPR spectrometer (E-Scan-Bruker BioSpin, GmbH).

For each recruited patient, the ROS production rate was determined using a recently implemented EPR method^24,26^. Briefly: 50 μL of each plasma sample was immediately treated with dissolved CMH (1-hydroxy-3-methoxycarbonyl-2,2,5,5-tetramethylpyrrolidine) probe (1:1). 50 *μ*L of the obtained solution was put in a glass EPR capillary tube, in turn placed inside the cavity of the E-scan spectrometer for data acquisition (microwave frequency 9.652 GHz; modulation frequency 86 kHz; modulation amplitude 2.28 G; sweep width 60 G, microwave power 21.90 mW, number of scans 10; receiver gain 3.17.10^1^). The sample temperature was stabilized and kept at 37 °C by the Temperature & Gas Controller “Bio III” unit, interfaced to the spectrometer.

All data were converted in absolute concentration levels (μmol· min^−1^) by adopting the CP (3-Carboxy-2,2,5,5-tetramethyl-1-pyrrolidinyloxy) stable radical as the external reference. The high reproducibility of the EPR measurements was well demonstrated previously^24^.

### HPLC analysis of erythrocytes glutathione

Total glutathione (GSH) was measured in erythrocytes by high-performance liquid chromatography, as previously described^27^. Values are expressed in μmol· L^−1^.

### Lipid peroxidation

Lipid peroxidation was measured in plasma spectrophotometrically at 532 nm (Infinite M200, Tecan) by adopting the thiobarbituric acid-reactive substances (TBARS) assay kit (Cayman Chemical).

A linear calibration curve was calculated from differently concentrated pure malon-di-aldehyde (MDA) solutions.

### Protein oxidation

A PC assay kit (Cayman Chemical) was used to colorimetrically evaluate in plasma the oxidized protein amount at 370 nm (Infinite M200, Tecan). The obtained values were normalized to the total protein concentration in the final pellet (280 nm), to take into account the protein loss during the washing steps.

### Antioxidant capacity

Total antioxidant capacity (TAC) levels (mM) in plasma were estimated using Antioxidant Assay Kit (Cayman Chemical) following the manufacturer’s protocol. A Trolox standard curve was used to quantitate the antioxidant capacity of the sample, measured in millimolar Trolox equivalents at 750 nm (Infinite M200, Tecan).

### Nitric oxides metabolites in plasma

Plasma EDTA samples were ultra-filtered through a 10 kDa molecular weight cut-off filter using an ultra-centrifuge at 14,000 × *g* for 60 min at 4 °C to reduce background absorbance due to the presence of hemoglobin. The ultra-filtered material was recovered and used to measure nitrite and nitrate concentrations using a commercial kit (Cayman Chemical). The samples were read by adding Griess reagents in at 545 nm. A linear calibration curve was computed from pure nitrite and nitrate standard.

### Statistical analysis

Statistical analysis was performed using GraphPad Prism package (GraphPad Prism 7, GraphPad Software Inc., San Diego, CA, USA).

For all parameters, descriptive statistics were calculated and the normality of the distribution was checked by the Shapiro-Wilk normality test. Data are presented as mean ± SD, 95% CI. Comparisons between experimental data were analyzed using analysis of variance ANOVA with a Bonferroni post-hoc test. Spearman’s correlation coefficient test (*r*, 95% confidence interval (CI)) was used for identifying relationships among selected parameters.

The diagnostic performance of individual assays was evaluated using receiver operating characteristic (ROC) curve analysis. A *p* < 0.05 value was considered statistically significant. The prospective power calculation to determine the significant experimental number was made by using the Freeware G*Power software (http://www.psycho.uniduesseldorf.de/abteilungen/aap/gpower3/). At a power of 80%, the calculated number of significant subjects was 11, well below the population of subjects recruited for this study.

## Results

Fifty-four CD patients (44 females; mean age 44 ± 13 years, range 19–80 years) were enrolled. In detail: 17 N-CD, 18 CD-GFD, and 19 NRCD.

Demographic and hematologic results are reported in Table 1. Age, gender, and GFD were not statistically different among the three groups. The investigated blood parameters, excluding leukocyte and platelet counts, were significantly lower in N-CD and NRCD compared to CD-GFD patients (*p* < 0.05).

**Table 1:** Clinical, demographic, and biochemical parameters of enrolled patients

	N-CD	CD-GFD	NRCD
*N* (m/f)	17 (5/11)	18 (1/17)	19 (4/15)
Age (years)	38.8 ± 10.4	43.1 ± 10.2	49.3 ± 16.6
Marsh-Oberhuber score			
0	0	13	0
1	0	5	0
2	0	0	2
3a	6	0	10
3b	8	0	2
3c	3	0	5
GFD duration (months)	Not applicable	117 ± 84	86 ± 82
Leukocytes (19^9^/L)	6.9 ± 1.6	6.3 ± 1.4	6.0 ± 1.2
Erythrocytes (10^12^/L)	4.2 ± 0.2	4.6 ± 0.6*	4.2 ± 0.3*
Hemoglobin Hb (g/dL)	12.8 ± 1.9	14.3 ± 1.1*	12.9 ± 1.1*
Hematocrit Hct (%)	38.6 ± 4.1	42.4 ± 4.3*	39.6 ± 2.4*
Corpuscular volume (fL)	82.7 ± 8.1	89.1 ± 6.4*	86.8 ± 7.1
MCH (pg)	28.1 ± 2.5	31.1 ± 1.7***	29.4 ± 2.1*
MCHC (g/dL)	32.7 ± 1.5	33.9 ± 0.1*	32.8 ± 1.1*
RDW (%)	13.1 ± 1.2	14.7 ± 1.1***	13.5 ± 1.2***
Platelets (10^9^/L)	271.2 ± 66.8	281.5 ± 44.1	305.0 ± 54.7

Data expressed as mean ± SD.

*CD* celiac disease, *GFD* gluten free diet, *MCH* Mean corpuscular hemoglobin, *MCHC* mean corpuscular hemoglobin concentration, *N-CD* naïve celiac patients, *CD-GFD* GFD-responsive patients, *NRCD* non-responsive CD, *RDW* red blood cell distribution width.

Significant differences compared to N-CD: *p < 0.05, ***p < 0.001.

**Fig. 1:**
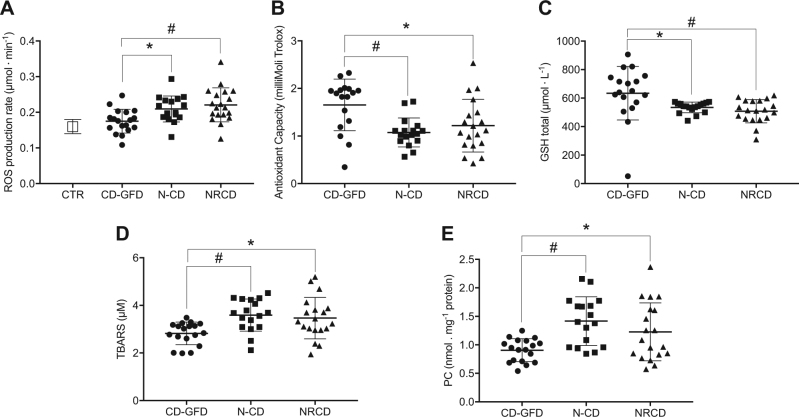
Scatter plots of oxidative stress biomarkers. **A** ROS production rate (μmol· min^−1^) detected by EPR technique; **B** total antioxidant capacity (TAC, mmol·equiv·Trolox/L); **C** total glutathione (GSH, μmol· L^−1^) concentrations in erythrocytes; **D** thiobarbituric acid-reactive substances (TBARS, μM); **E** protein carbonyl (PC, nmol. mg^−1^ protein) in plasma of N-CD (*n* = 17, square symbol), CD-GFD (*n* = 18, circle symbol) and NRCD (*n* = 19, triangle symbol) patients and in healthy subjects as controls (CTR, the empty square, *n* = 100) evaluated in Mrakic-Sposta S. et al., 2014. The results are expressed as mean ± SD. Significant differences: *p < 0.05; ^#^*p* < 0.01

As shown in Fig. 1A, plasmatic ROS production is significantly increased in N-CD (0.21 ± 0.03 μmol· min^−1^) and NRCD (0.22 ± 0.04 μmol·min^−1^) patients compared with CD-GFD (0.17 ± 0.03 μmol·min^−1^). Moreover, no significant difference was found between CD-GFD and CTR (0.17 ± 0.03, vs 0.16 ± 0.02 μmol· min^−1^ respectively)^24^.

Plasmatic TAC levels were significantly lower in the N-CD and NRCD groups compared with CD-GFD (1.07 ± 0.30 mM *vs* 1.16 ± 0.47 mM *vs* 1.68 ± 0.54 mM in N-CD, NRCD, and CD-GFD respectively) (Fig. 1B). Similarly, GSH levels in erythrocytes were significantly lower in the N-CD and NRCD (Fig. 1C) compared with CD-GFD (534.40 ± 37.46 μmol· L^−1^ vs 507.80 ± 81.73 μmol· L^−1^ vs 634.00 ± 187.80 μmol· L^−1^ in N-CD, NRCD, and CD-GFD respectively).

Oxidative damage in plasma was assessed by TBARS and PC determination: Fig. 1D shows significantly higher values of TBARS in the N-CD and NRCD groups compared to CD-GFD (3.59 ± 0.67 μM vs 3.46 ± 0.87 μM vs 2.82 ± 0.47 μM in N-CD, NRCD, and CD-GFD respectively). Accumulation of PC (Fig. 1E) was significantly higher in the N-CD and NRCD compared to CD-GFD (1.42 ± 0.43 nmol. mg^−1^ protein vs 1.23 ± 0.53 nmol. mg^−1^ protein vs 0.91 ± 0.20 nmol. mg^−1^ protein in N-CD, NRCD, and CD-GFD respectively).

**Fig. 2:**
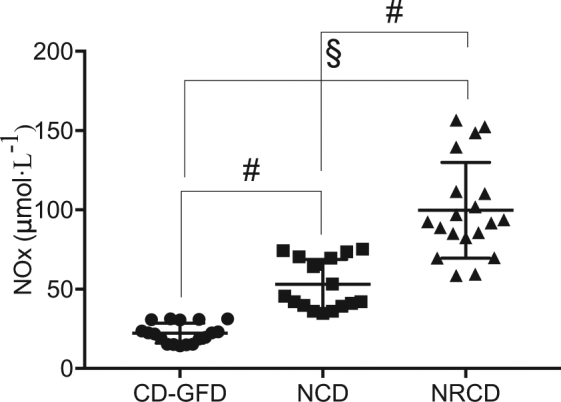
Scatter plots of the plasma nitrate concentration (μmol· L^−1^) in N-CD (*n* = 17, square symbol), CD-GFD (*n* = 18, circle symbol), and NRCD (*n* = 19, triangle symbol) patients. The results are expressed as mean ± SD. Significant differences: ^#^*p* < 0.01 and ^§^*p* < 0.001

In Fig. 2 data of plasmatic nitrates concentrations (NO_*x*_ = NO_2_ + NO_3_) are reported. Significantly higher values were recorded for the NRCD and N-CD compared to CD-GFD (99.74 ± 30.76 μmol· L^−1^ vs 54.61 ± 14.57 μmol· L^−1^ vs 22.21 ± 6.92 μmol· L^−1^ in NRCD, N-CD, and CD-GFD respectively) and a significantly different NOx concentration between N-CD and NR-CD was recorded too.

**Fig. 3:**
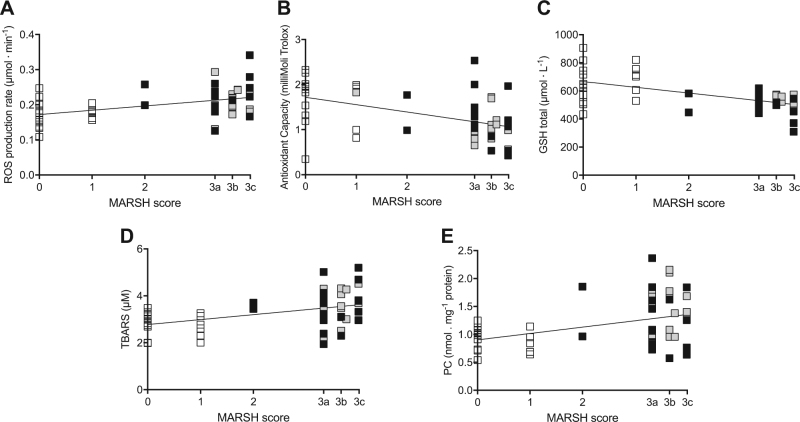
The relationship between the Marsh score and oxidative stress biomarkers. **A** ROS production rate (μmol· min^−1^), **B** TAC (mmol.equiv.Trolox.L^−1^), **C** GSH (μmol·L^−1^), **D** TBARS (μM) and (**E**) PC (nmol. mg^−1^ protein) assessed in the plasma of N-CD (*n* = 17, white symbol), CD-GFD (*n* = 18, gray symbol) and NRCD (*n* = 19, black symbol) patients. The linear regression fit (solid line) is reported for each relationship

The possible correlation between the classification of histologic findings in CD (Marsh grade), plasmatic ROS production and OxS biomarkers was investigated for all the groups (CD-GFD, N-CD, and NRCD) (Fig. 3).

A significant direct relationship was found between the Marsh score and ROS production rate (*R*^2^ = 0.19; *p* < 0.0009), TBARS (*R*^2^ = 0.19; *p* < 0.0001), and PC (*R*^2^ = 0.16; *p* < 0.002) (Fig. 3A,D,E). Contrariwise, an inverse correlation was found between the Marsh grade and TAC (*R*^2^ = 0.22; *p* < 0.0003) and GSH (*R*^2^ = 0.34; *p* < 0.0001) (Fig. 3B, C).

**Fig. 4:**
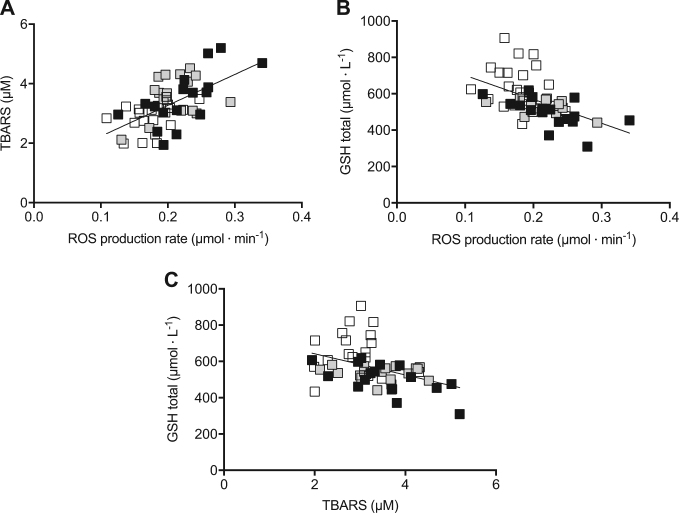
The relationship between selected oxidative stress biomarkers. **A** TBARS (μM), **B** GSH (μmol· L^−1^), and (**C**) between TBARS (μM) and GSH (μmol· L^−1^) in the plasma of N-CD (*n* = 17, white symbol), CD-GFD (*n* = 18, gray symbol) and NRCD (*n* = 19, black symbol) patients. The linear regression fit (solid line) is reported for each relationship

For all the three groups of patients, a correlation between ROS production, GSH in erythrocytes and TBARS concentrations was found (Fig. 4). A positive relationship was found between ROS production and TBARS concentration (*R*^2^ = 0.36; *p* < 0.0001) (Fig. 4a), while an inverse relationship was found with GSH concentration (*R*^2^ = 0.27; *p* < 0.0001) (Fig. 4b). At high ROS production rate levels corresponded greater TBARS concentrations and lower GSH levels.

Moreover, a positive relationship was found between TBARS concentrations and GSH levels (*R*^2^ = 0.16; *p* < 0.001) (Fig. 4C), as a correlation between OxS biomarkers and hematological data for the N-CD and NRCD groups (no correlation was found in CD-GFD group). Table 2 reports the significant correlation in both groups of CD patents.

**Table 2:** Relationships between the OxS biomarkers and hematologic data of the patients affected by CD

		*R* ^2^	*P*	
N-CD	Hemoglobin vs	TAC	0.22	0.05
		Leukocytes	0.35	0.01
		Erythrocytes	0.26	0.03
		Hematocrit	0.27	0.03
		Corpuscular volume	0.64	0.001
		MCH	0.30	0.02
	Hematocrit vs	Hemoglobin	0.26	0.03
		Corpuscular volume	0.29	0.02
		MCH	0.30	0.02
NRCD	Hemoglobin vs	Corpuscular volume	0.44	0.002
		MCHC	0.26	0.02
	Hematocrit vs	Total GSH	0.22	0.04

Finally, we analyzed the ROC curves of OxS markers to discriminate duodenal atrophy (i.e., Marsh 3 lesions) and of ROS to discriminate the different CD types (CD-GFD, N-CD, and NRCD). ROS showed a good area under the curve (>0.7) to discriminate atrophy and NRCD (see supplementary figure 1).

## Discussion

Our study demonstrates the impairment of oxidative balance in CD patients, both treated and untreated, and the potential use of OxS biomarkers in clinical practice during follow-up. The main purpose of this study was to describe the oxidative profile of celiac patients, especially of those not responding to GFD.

There is a growing number of studies in the literature showing how ROS are involved in the pathology of many diseases, including several gastrointestinal disorders.

CD is an autoimmune gastrointestinal disorder caused by gluten ingestion in genetically predisposed individuals leading to an atrophy of the duodenal mucosa. Its clinical presentation is heterogeneous and varies greatly with the age of patients, duration, and intensity of the disease and the possible presence of extra-intestinal disorders^28^. Inflammation and OxS due to an increase of ROS and a decrease of antioxidant defenses are involved in the molecular mechanisms of CD^18,29,30^.

Understanding the oxidative-inflammatory altered condition that characterizes NRCD patients is a critical achievement for the development of new medical (and pharmacological) approaches.

Currently, physicians are unable with serological biomarkers to distinguish patients with active or inactive CD during GFD. As invasive techniques are still required to differentiate between different forms of CD, the purpose of the present analysis has been to evaluate the ability of markers from peripheral blood to distinguish the various CD subsets.

For the first time, the present study shows a significant correlation between plasmatic ROS content, quantified with EPR direct method, and the disease histological grade (Marsh-Oberhuber) and type, especially NRCD, as demonstrated by ROC.

No significant difference was detected between celiac patients and healthy controls in respect to age and gender.

In CD iron deficiency anemia is primarily a consequence of the chronic mucosal damage and impairment of absorption mechanisms in the small bowel, due to the inflammatory process^31–33^. In our study hemoglobin values in CD and NRCD were in the normal range 12–15.6 g/dL, although there was a clear trend towards mild anemia. According to the results, it clearly appears that NRCD patients, in spite of the GFD therapy, have a hematologic framework comparable to N-CD patients, in whom the pathology was diagnosed for the first time.

The pathogenesis of CD has not been fully explained. The imbalance of OxS was demonstrated in the pathomechanism of gastrointestinal diseases, in particularly for CD in adults, and is also followed by an increased production of ROS^34^ and reduced antioxidant protection. Indeed Boda et al^35^. suggested that in patients with active CD, gluten ingestion, along with the resulting inflammation, causes the activation of xantine oxidase in enterocytes, which results in the overproduction of ROS and further damage to the intestinal mucosa. Moreover the results of various investigations suggest that gliadin disturbs the pro-oxidant-antioxidant balance in the small-intestinal mucosa of affected individuals through the overproduction of ROS^30,35^.

In our study analyzing the plasma levels of ROS production, a significant increase in adult N-CD and NRCD compared to CD-GFD patients was recorded. Significant decrease of the TAC levels in patients with naive CD (35%) and celiac patients not responding to a GFD (25%) as compared to celiac patients responder to GFD was observed too. This indicates an inadequate physiological response to a higher rate of production of ROS in active celiac patients and especially in NRCD patients, for whom the TAC is still compromised in spite of their GFD regimen. The lower plasmatic TAC in N-CD and especially in NRCD patients is probably the result of a malabsorption of dietary antioxidants due to the mucosal damage. In agreement with this hypothesis other authors have reported lower levels of antioxidants (retinol, α-tocopherol, and ascorbic acid) in the plasma of patients with active CD^29,36^.

The GSH redox cycle is the principal mechanism of detoxifying lipid hydroperoxides in the intestine^37^. High levels of cell GSH are preserved by *de-novo* synthesis, regeneration from glutathione disulfide, and in several cell types, including enterocytes, by an import via a Na^+^-dependent transport system^38^. Several *in-vitro* studies have reported on the pro-oxidative effects of gliadin in human intestinal cell cultures; Rivabene and colleagues^39^ showed that the antiproliferative effect of gliadin is associated with pro-oxidative changes, such as the high level of lipid hydroperoxides, reduction of GSH level and gradual disappearance of sulfhydryl groups in proteins. Elli et al. demonstrated that gliadin causes a reduction in the GSH content and a gliadin concentration-related decrease in the activity of glutathione reductase, glutathione peroxidase and GSH-S-transferase^5^.

Besides the intestinal mucosa, the erythrocytes are rich in glutathione and glutathione-related enzymes. In our study, a significant lower (23%) GSH concentration was found in the erythrocytes of N-CD and NRCD patients compared to control subjects. The low concentration of GSH in NRCD patients can be explained with the assumption that there is a decrease in glutathione peroxidase and glutathione reductase activities such as reported both in biopsies and erythrocytes from celiac patients with severe villous atrophy^40,41^.

It is very interesting to note how GSH concentration is also indirectly related to a clinical parameter such as Marsh score. Furthermore, in CD patients the ROS production resulted correlated positively with TBARS and negatively with GSH levels as previously reported in healthy subjects^24,42^. In this study for the first time we have demonstrated significant correlations between the histological grade (Marsh-Oberhuber) score and TAC (Fig. 3B) GSH (Fig. 3C), ROS production (Fig. 3a) and OxS biomarkers (TBARS: Fig. 3D; PC: Fig. 3E).

An increased plasma concentration of TBARS (23%) indicating a not adequate anti-oxidative system in N-CD and NRCD patients compared to CD-GFD subjects was found. The data are in agreement with those reported by Stojiljkovic et al^43^. on plasma lipid peroxidation biomarkers in active celiac patients.

The increased lipid peroxidation of plasma chylomicrons and low-density lipoproteins in a celiac patient has been also reported^44^. It was shown that a high lipid hydroperoxides level causes single- and double-strand DNA breaks as well as the oxidative damage of cell membranes^45^.

In our study, the increase of TBARS concentration correlated with the reduction of the GSH content. Elevated TBARS and the shift of the cellular pro-oxidant/antioxidant balance, is reported affect cell proliferation, differentiation or apoptotic responses and disrupt tissue homeostasis^37^. This may, in part, explain the increased risk of refractory CD and malignancy, observed in celiac patients that do not respond to a GFD.

The PC content is the most general and widely used marker of severe oxidative protein damage. In our study, we have found a significant increase: 51% in N-CD and 17% in NRCD patients compared to CD-GFD subjects. The data are consistent with those reported by Odetti et al^29^.

NO is a free radical gas, produced by the enzyme NO synthase (NOS), that has a wide variety of physiological and pathological roles in the gastrointestinal tract^21,46^. NO is rapidly metabolized by erythrocytes into the stable end-products nitrate and nitrite (NOx), which can be easily measured^21^.

Some authors have noted increased levels of NOx in the serum and urine of CD children, with a positive correlation between the concentration of NOx and increased concentration of iNOS in the small intestine^19,47,48^. Murray et al. have showed that plasmatic NOx concentrations in adult patients with CD are higher than in treated patients with GFD and those with other upper gastrointestinal disorders^21^. The higher production of NO metabolites and the consequent nitrosative stress promote the impairment of tight junctions in the small intestine of CD patients, perhaps by down-regulating the expression of zona-ocludens-1^49^.

The results of our study, where NRCD patients exhibited high levels of NOx compared to the control condition and showed severe histologic changes too, are similar to data reported previously^21,22^. Moreover, ROC analysis confirmed the usefullness fo ROS in discriminating the NRCD from the responsovise CD wirg represents a diagnostic dillemma.

## Conclusions

Our study investigated the possible correlations in all the patient groups of OxS biomarkers with the Marsh-score classified histologic findings in CD.

For the first time we have shown how the increase in the ROS production rate, assessed in the peripheral blood, is in strict correlation with the severity of intestinal damage, evaluated by histological analysis (Marsh score).

Moreover our results confirm that OxS is strongly associated with gluten-related disorders and an important factor in the pathogenesis of CD so it may be an important additional parameter for monitoring a gluten-related disorder and for establishing the effectiveness of GFD.

## Study Highlights

### What is current knowledge

- Oxidative stress is involved in the damage of cellular lipids, proteins and DNA, increased cellular swelling and decreased cell membrane fluidity
- Oxidative system imbalance plays a pivotal role in the pathogenesis of different gastrointestinal disorders.
- In celiac disease, an impairment of oxidative balance has been demonstrated after gluten exposure.

### What is new here

- An alteration of oxidative stress biomarkers has been found in naïve and non responsive celiac patients compared to responsive patients.
- A significant correlation between the severity of duodenal atrophy and reactive oxygen species have been found.
- The alterations of oxidative stress biomarkers and their correlation with the intestinal damage could be useful as indicators of celiac disease activity

## Electronic supplementary material

Supplementary Figure 1
