# Supplementary material for: Oxidative stress as a biomarker for monitoring treated celiac disease
Source: Clin Transl Gastroenterol. 2018 Jun 8;9(6):157. doi: 10.1038/s41424-018-0031-6 (PMC5992147; doi:10.1038/s41424-018-0031-6)
Supplement: Supplementary file 1 — Supplementary Figure 1 [file 41424_2018_31_MOESM1_ESM.pdf]

Supplemetary Figure 1

ROC curves and statistical analysis of the different oxidative stress markers to discriminate duodenal atrophy (Marsh 3) (A) and of ROS to individuate CD-GFD (B), N-CD (C) and NRCD (D) groups

A

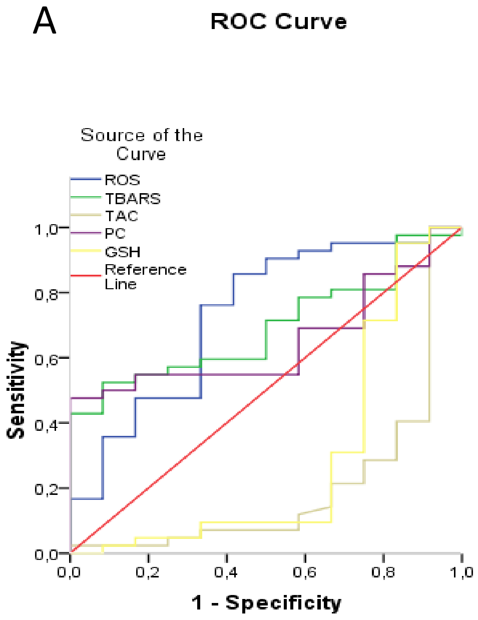

Variable	Area	Std. Error[a]	Asymptotic 95% Confidence Interval	
			Lower Bound	Upper Bound
ROS	,732	,087	,562	,903
TBARS	,694	,073	,551	,838
TAC	,197	,084	,033	,362
PC	,653	,076	,505	,801
GSH	,290	,107	,079	,500

B

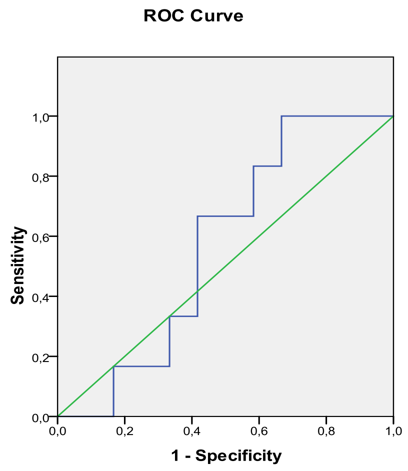

Area	Std. Error[a]	Asymptotic 95% Confidence Interval	
		Lower Bound	Upper Bound
,569	,135	,305	,834

C

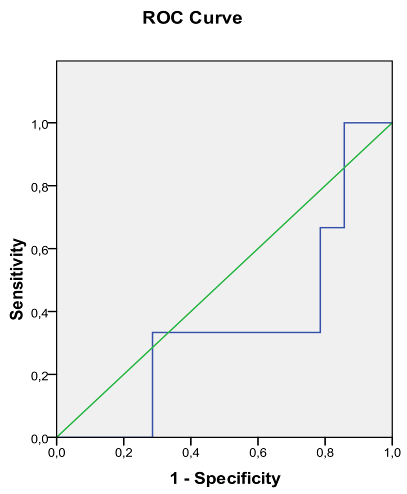

Area	Std. Error[a]	Asymptotic 95% Confidence Interval	
		Lower Bound	Upper Bound
,357	,174	,016	,699

D

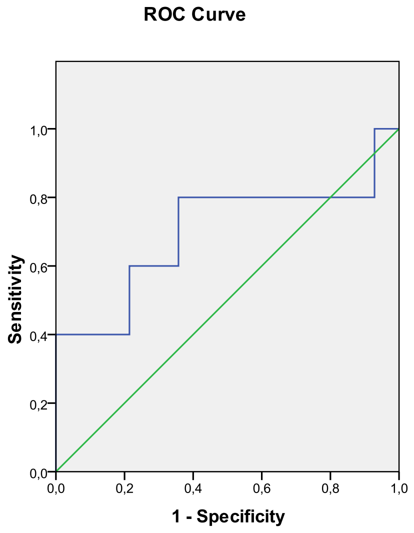

Area	Std. Error[a]	Asymptotic 95% Confidence Interval	
		Lower Bound	Upper Bound
,700	,163	,354	1,000
